# Generalist-to-specialist diagnostic consultations in a tertiary hospital: an outcome-based retrospective observational study

**DOI:** 10.1186/s12913-026-15278-4

**Published:** 2026-08-01

**Authors:** Ren Kawamura, Yukinori Harada, Yudai Suzuki, Yumi Otaka, Shogo Hanai, Shinichi Katsukura, Masashi Yokose, Tetsu Sakamoto, Taro Shimizu

**Affiliations:** 1 Department of Diagnostic and Generalist Medicine, Dokkyo Medical University Hospital, 880 Kitakobayashi, Mibu, Shimotsuga, Tochigi 321-0293 Japan; 2 Department of Internal Medicine, Yamagata Prefectural Kahoku Hospital, Yamagata, Japan; 3 Department of Infectious Diseases, Fujita Health University, Toyoake, Aichi Japan

**Keywords:** General internal medicine, Specialty departments, Generalists, Specialists, Consultations, Diagnosis

## Abstract

**Background:**

Consultations between the General Internal Medicine (GIM) and specialty departments are becoming increasingly crucial to achieving team-based diagnosis and diagnostic excellence; however, there are limited studies on the appropriateness of GIM consultations with specialties. This study aimed to evaluate the proportion of diagnostic consultations from GIM to specialty departments that fulfilled pre-specified, outcome-based criteria for contributing to the establishment of a correct final diagnosis and to identify factors associated with consultations not meeting these criteria.

**Methods:**

This retrospective, single-center observational study was conducted at a university hospital in Japan. Consultations from the GIM to specialty departments involving patients aged 15 years and older between January 1, 2021 and December 31, 2021 were analysed. The primary outcome was the proportion of diagnostic consultations fulfilling these pre-specified, outcome-based criteria. Factors associated with consultations not meeting these criteria were also analysed.

**Results:**

In total, 610 consultations for 454 patients were included. Diagnostic consultations accounted for 352 cases (57.7%), among which 229 cases (65.1%) fulfilled these criteria, while 123 cases (34.9%) did not. Consultations not meeting these criteria were associated with the following factors: difficulty in diagnosis at the time of consultation because of undifferentiated symptoms and findings in 41 cases (33.3%), inadequate test implementation and interpretation in 39 cases (31.7%), and insufficient patient information in 12 cases (9.8%).

**Conclusion:**

Approximately 30% of diagnostic consultations from GIM to specialty departments did not fulfill these pre-specified, outcome-based criteria. Among consultations not meeting these criteria, potentially modifiable factors included inadequate diagnostic test implementation or interpretation and insufficient patient information, whereas approximately one-third involved diagnostic difficulty at the time of consultation because of undifferentiated symptoms and findings. Future efforts should focus on improving diagnostic test implementation and interpretation and on identifying diagnostically difficult cases that can be safely managed by GIM with continued assessment before immediate specialist consultation.

## Background

Pursuing diagnostic excellence, defined as “making a correct and timely diagnosis using the fewest resources while maximizing patient experience and managing uncertainty,” is considered an essential goal for high-quality medical practice [1]. It includes safe, effective, patient-centered, timely, efficient, and equitable care [2]. Furthermore, it has been suggested that diagnostic excellence requires a team-based approach rather than the judgment of a single clinician; general internal medicine (GIM) practitioners can play a central role in this approach [1, 3]. Consequently, consultations and referrals between the GIM and specialty departments have become increasingly necessary.

Prior studies have analysed the outcomes of consultations from specialty departments to the GIM department, showing that the GIM department can function as a diagnostic hub for specialty departments [4, 5]. However, GIM departments often need to consult specialty departments to establish a correct final diagnosis by performing special tests or diagnostic interventions. Previous studies have shown that generalists request fewer unnecessary consultations than do specialists [6]. Nevertheless, the quality and appropriateness of specialty consultations still require improvement, as suboptimal specialty consultations have been reported as a factor in straining medical resources and contributing to diagnostic errors[7]. In addition, it is necessary to reduce the burden on specialists through appropriate consultations in situations where the number of specialists is expected to decrease [8].

Two studies, conducted at university hospitals in Japan and the US, reported that approximately 30% of outpatient diagnostic consultations from the GIM department were inappropriate [9, 10]. However, neither study examined the factors associated with such consultations. In one prior study of acute-phase inpatients in tertiary medical institutions, approximately 50% of the patients required consultations from the GIM department to the specialty departments during their hospitalization [11]. However, data on the aim and quality of these consultations are lacking. Therefore, to achieve diagnostic excellence, it is necessary to understand the proportion of diagnostic consultations that may have room for improvement and to identify factors associated with them.

The present study aimed to evaluate the proportion of diagnostic consultations from the GIM to other specialty departments that fulfilled pre-specified, outcome-based criteria for contributing to the establishment of a correct final diagnosis and to identify factors associated with consultations not meeting these criteria.

## Methods

### Ethical consideration

This study complied with the Declaration of Helsinki and was approved by the university hospital’s Research Ethics Committee (R-62–16 J). All data were anonymized and aggregated; therefore, the requirement for written informed consent was waived by the Research Ethics Committee because we used an opt-out method. Patients were informed about the study by providing detailed information in the outpatient waiting areas and inpatient wards of the Department of Diagnostic and Generalist Medicine (DGM).

### Study design and setting

This single-center, retrospective, observational study used data from a university hospital with 29 departments in Japan. The DGM in this hospital functions as a hospital-based generalist department and covers roles commonly performed by GIM departments in Japan, including outpatient care, inpatient care, and emergency medicine. Outpatient care includes referrals from within and outside the hospital, as well as walk-in first visits to internal medicine. The DGM provides outpatient and inpatient care for diagnostically difficult or undifferentiated cases.

### Patient population

We included consultations from the DGM to other specialty departments within the university hospital for outpatients and hospitalized patients aged 15 years and older between January 1, 2021 and December 31, 2021. We excluded patients who refused to participate in the study using the opt-out method and those who met the following criteria to focus on consultations for only diagnostic purposes: (1) inpatients’ consultations to the Department of Rehabilitation to initiate or modify rehabilitation or for requesting rehabilitation summaries; (2) inpatient consultations to the Department of Nephrology and Hypertension for continued inpatient dialysis or plasma exchange or for requesting dialysis summaries; (3) consultations to the Department of Oral and Maxillofacial Surgery for oral evaluation, oral care, or denture adjustment; (4) duplicate consultations with identical content; (5) consultations to the Department of Cardiovascular Medicine for echocardiography or pacemaker adjustments; (6) consultations to the Department of Endocrinology and Metabolism for blood glucose management during hospitalization of patients who were followed-up as outpatients by the department before hospitalization; (7) consultations in which the patient was not actually seen; and (8) consultations where the patient declined to undergo the test or discontinued the visit.

### Data collection

We retrospectively reviewed patients’ medical records and extracted data regarding age, sex, consulting location (outpatient or inpatient), name of the specialty department, consultation date, chief complaint, contents of the consultation request, and contents of the consultation reply. Notably, all cases were independently reviewed by two of the eight reviewers.

The medical records were reviewed as follows: First, to focus on consultations for diagnostic purposes, we excluded patients who met the exclusion criteria mentioned above based on the contents of the consultation request. Next, the purpose of the consultation was classified into one of the following categories: (1) diagnosis with strong suspicion, (2) other diagnoses, (3) tests, (4) treatment, (5) management, and (6) others. These classification categories were created based on previous studies [9, 10, 12, 13]. Consultation for a “diagnosis with strong suspicion” was defined as a consultation for diagnosis when a specific disease was strongly suspected. Consultation for “other diagnoses” was defined as any other consultation for a diagnosis. When tests were requested, the consultation was classified as “for diagnosis with strong suspicion” or “for other diagnoses,” if possible. The consultation was classified as “tests” if it could not be classified in either category. “Treatment” was defined as a consultation to request treatment for a newly diagnosed disease, whereas “management” was defined as a consultation for additional treatment or management of an existing disease. Table 1 presents specific examples of the classification of consultation purposes.

**Table 1:** Classification of the consultation purposes

Age, Sex	Chief complaint	Specialty department	Reason for consultation	Purpose
50s, F	Generalized pain	Rheumatology	Polymyalgia rheumatica was suspected based on the physical findings and test results	Diagnosis with strong suspicion
60s, M	Muscle weakness in the lower limbs	Neurology	Diagnosis could not be narrowed down to a specific disease; however, some form of neuromuscular disease was suspected.	Other diagnoses
60s, M	Bilateral tinnitus	Otorhinolaryngology, Head and Neck Surgery	A hearing test was requested due to complaints of left hearing loss and bilateral tinnitus.	Tests
90s, F	Vomiting	Surgical Oncology	A computed tomography scan revealed adhesive intestinal obstruction, and surgical consultation was performed for treatment.	Treatment
70s, F	Panic attack	Neuropsychiatry	The patient attended the GIM department with a diagnosis of panic disorder, but specialist management was considered necessary due to worsening symptoms.	Management
10s, F	Hemoptysis	Thoracic Surgery	The consultation was conducted to obtain a specialist’s opinion on whether the nodule in the upper mediastinum near the bronchus, observed on computed tomography, could cause hemoptysis.	Others

Abbreviations: GIM, general internal medicine; F, female; M, male

If a “diagnosis with strong suspicion” was applicable, the disease name assumed by the DGM was extracted from the contents of the consultation request. Next, we evaluated whether a correct final diagnosis was made in the initially consulted specialty department. If there was a correct final diagnosis, the final diagnosis and whether it was included in the consultation request was assessed. If a correct final diagnosis was not made by the initial specialty department, we subsequently assessed whether a correct final diagnosis was made by the DGM or an additional consulting specialty department. The period from the first visit to the DGM to the day the correct final diagnosis was made was also assessed.

If “other diagnoses” were applicable, the name of the diseases, as assumed by the DGM, was similarly extracted. Subsequently, as described above, the presence or absence of a correct final diagnosis in the consulted specialty department was extracted. The same items for the “diagnosis with strong suspicion” were extracted based on the presence or absence of a correct final diagnosis.

No further information was collected regarding consultations other than those for diagnostic purposes. All cases were independently reviewed by two reviewers. The initial classifications by the two reviewers were used to calculate inter-rater agreement. When disagreements occurred, the two reviewers re-examined the consultation request, consultation reply, and subsequent clinical course, and the final classification was determined through discussion. Because downstream assessments were performed only when a reviewer classified the consultation purpose as diagnostic, some downstream variables were structurally not applicable when that reviewer classified the consultation purpose as non-diagnostic.

### Study-defined outcome-based criteria for diagnostic consultations

Because no universally accepted criteria exist for retrospectively determining the clinical appropriateness of diagnostic consultations, we developed operational, outcome-based study-defined criteria for diagnostic consultations. These criteria were intended to identify consultations that contributed to establishing a correct final diagnosis in the initially consulted specialty department and to explore factors associated with consultations not meeting these criteria. They were not intended to determine whether each consultation was clinically justified at the time it was requested.

A diagnostic consultation was considered to have met the study-defined criteria as follows:

1. Diagnosis with strong suspicion: consultations in which the correct final diagnosis was made in the initially consulted specialty department and the name of the correct final diagnosis was included in the DGM consultation request.
2. Other diagnoses: consultations that led to a correct final diagnosis in the initially consulted specialty department.

For consultations classified as “diagnosis with strong suspicion,” we required that the final diagnosis be explicitly included in the DGM consultation request because these consultations were based on a specific suspected disease at the time of referral. In contrast, for consultations classified as “other diagnoses,” we did not require that the final diagnosis be listed in the request because these consultations were made in broader or less differentiated diagnostic contexts.

Diagnostic consultations that did not meet the above criteria were classified as consultations not meeting the study-defined criteria. For these consultations, we evaluated factors associated with not meeting the criteria, including inadequate patient information collection, inadequate test implementation or interpretation, difficulty in diagnosis at the time of consultation because of undifferentiated symptoms and findings, and others. The “others” category was used when the consultation did not meet the study-defined criteria but could not be categorized into the predefined factors or when reviewers considered the consultation clinically justified despite not meeting the outcome-based criteria.

### Outcomes

The primary outcome was the proportion of diagnostic consultations meeting the study-defined criteria. Secondary outcomes included consultation purposes and factors associated with consultations not meeting the study-defined criteria.

### Sample size calculation

Based on previous studies 9, 10 we estimated that the rate of diagnostic consultation would be 40% and that the proportion of diagnostic consultations meeting the study-defined criteria would be 70%. We thus calculated the required sample size to be 333 cases, with four independent variables set for logistic regression analysis.

### Statistical analysis

Continuous data are presented as medians with interquartile ranges. Categorical data are presented as counts and proportions (%). For baseline characteristics, we used chi-square tests for sex and consultation purpose comparisons between outpatients and inpatients. We used the unpaired t-test for group comparisons by age. We further used the unpaired t-test for intergroup comparisons of the days between the first visit and correct final diagnosis between outpatients and inpatients. Cohen’s kappa was calculated to examine inter-rater agreement for the purpose of consultation between the two reviewers at the initial review. Because downstream assessments were performed only for consultations classified as diagnostic by each reviewer, inter-rater agreement for whether consultations met the study-defined criteria was calculated among consultations that both reviewers initially classified as diagnostic consultations and for which both reviewers provided this assessment. Inter-rater agreement for factors associated with consultations not meeting the study-defined criteria was calculated among consultations for which both reviewers judged that the study-defined criteria were not met and provided a reason classification. We additionally calculated the proportion of diagnostic consultations meeting the study-defined criteria with 95% confidence intervals (CIs) for the primary outcome. Sensitivity analyses were performed by reclassifying consultations that did not meet the study-defined criteria but were considered clinically justified by the reviewers as consultations meeting the criteria. An additional exploratory sensitivity analysis was performed to assess the impact of consultations categorized as difficult to diagnose at the time of consultation because of undifferentiated symptoms and findings. We used chi-square tests to compare the proportion of consultations meeting the study-defined criteria between “diagnosis with strong suspicion” and “other diagnoses.” We used binary logistic regression analysis, considering whether consultations met the study-defined criteria as the outcome variable and age, sex, location, and degree of diagnostic certainty as exposure variables. P-values were based on two-tailed statistical tests, and statistical significance was set at *P* < 0.05. All statistical analyses were conducted using STATA statistical software version 18.0 SE (StataCorp).

## Results

**Fig. 1:**
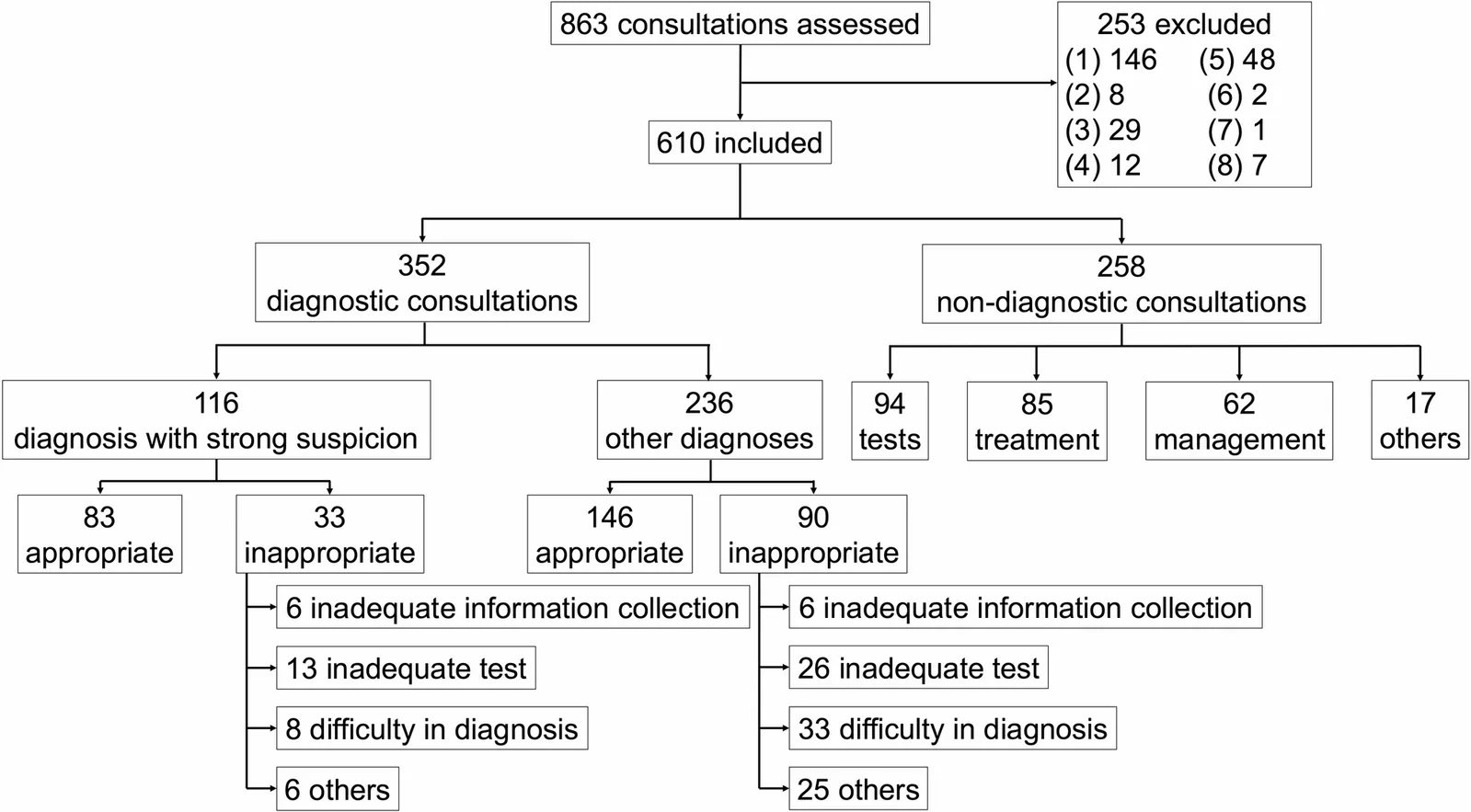
Study flowchart. The exclusion criteria are as follows: (1) inpatient consultations to the Department of Rehabilitation to initiate or modify rehabilitation or for requesting rehabilitation summaries; (2) inpatient consultations to the Department of Nephrology and Hypertension for continued inpatient dialysis or plasma exchange or for requesting dialysis summaries; (3) consultations to the Department of Oral and Maxillofacial Surgery for oral evaluation, oral care, or denture adjustment; (4) duplicate consultations with identical content; (5) consultations to the Department of Cardiovascular Medicine for echocardiography or pacemaker adjustments; (6) consultations to the Department of Endocrinology and Metabolism for blood glucose management during hospitalization of patients who were followed-up as outpatients by the department before hospitalization; (7) consultations in which the patient was not actually seen; and (8) consultations where the patient declined to undergo the test or discontinued the visit

In total, 863 consultations involving 513 patients were conducted during the study period. After excluding 253 consultations that met the exclusion criteria, 610 consultations involving 454 patients were included in the analysis (Fig. 1). The median age of the patients was 64 years (interquartile range [IQR], 49–74), and 215 (47.4%) patients were male. Other baseline characteristics are presented in Table 2.

**Table 2:** Baseline characteristics and the breakdown of consultation purposes

	Total (*N* = 610)	Outpatient (*n* = 441)	Inpatient (*n* = 169)	*P*-value
Age in years, median (IQR) Age in years, mean ± SD	64 (49–74)	61 (46–73) 58.2 ± 19.8	70 (60–80) 67.5 ± 17.3	< 0.01*
Males, n (%)	215 (47.4)	171 (47.2)	44 (47.8)	0.92†
Purpose, n (%) Diagnosis with strong suspicion Other diagnoses Tests Treatment Management Others	116 (19.0) 236 (38.7) 94 (15.4) 85 (13.9) 62 (10.2) 17 (2.8)	102 (23.1) 175 (39.7) 53 (12.0) 68 (15.4) 34 (7.7) 9 (2.0)	14 (8.3) 61 (36.1) 41 (24.3) 17 (10.1) 28 (16.6) 8 (4.7)	< 0.01†

* The t-test was performed based on the mean values; however, due to the non-normality of data, the median and IQR of age are also reported for a more comprehensive understanding of the distribution

† Chi-square tests were conducted to compare sex and consultation purposes between outpatients and inpatients

Abbreviations: IQR, interquartile range; SD, standard deviation

**Fig. 2:**
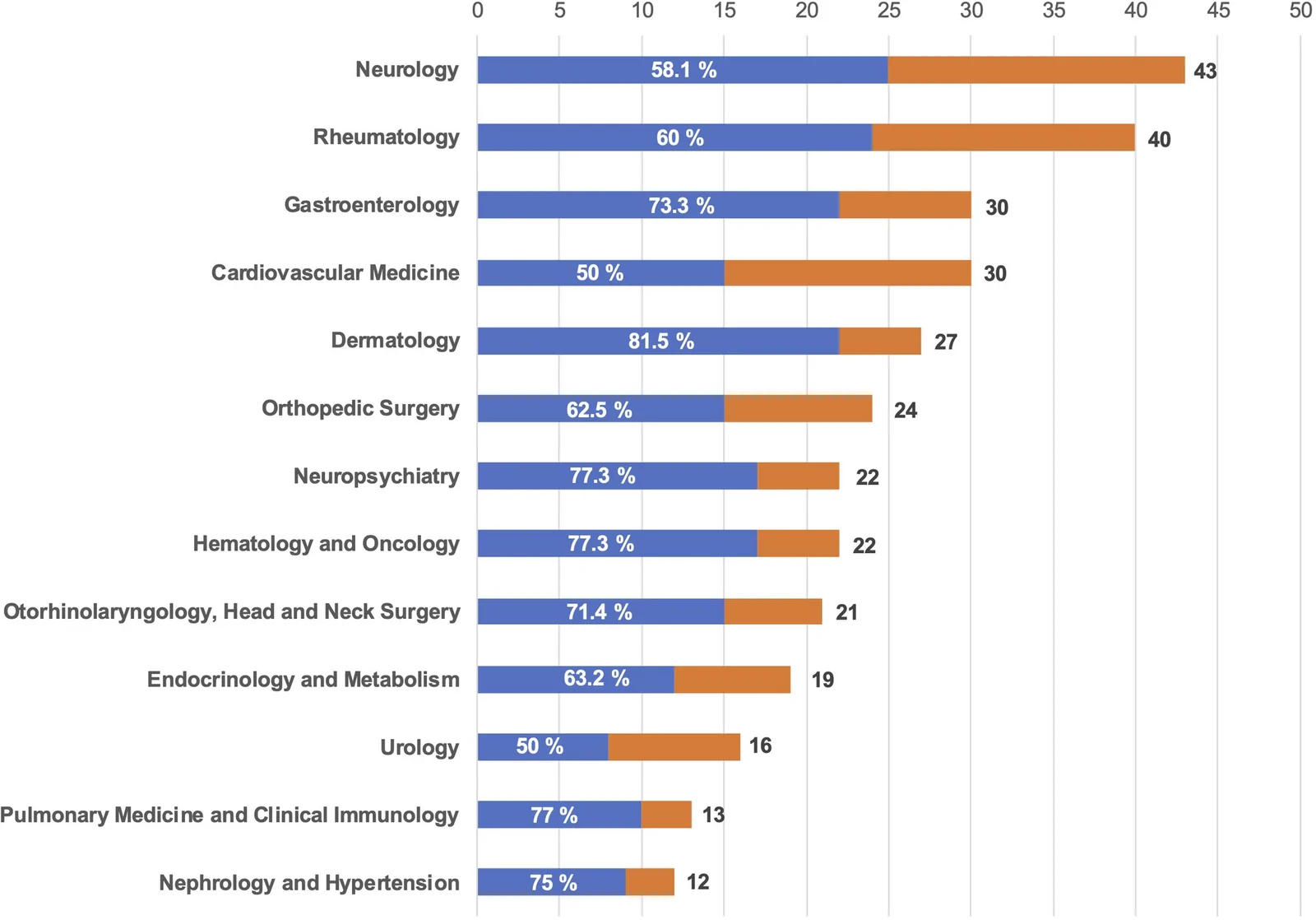
Number of diagnostic consultations and consultations meeting the study-defined criteria by specialty department. The numbers outside the horizontal bars represent the total number of diagnostic consultations. The blue scale indicates the number of consultations meeting the study-defined criteria. The proportion of consultations meeting the study-defined criteria is also shown

Overall, diagnostic consultations comprised 57.7% (352/610) of consultations from the DGM to other specialty departments: diagnosis with strong suspicion 116/610 (19.0%) and other diagnoses 236/610 (38.7%). Other reasons for consultations were tests in 94 cases (15.4%), treatment in 85 cases (13.9%), management in 62 cases (10.2%), and others in 17 cases (2.8%). The inter-rater agreement on the purpose of the consultation at the initial review was 53.3% (325/610; Cohen’s kappa = 0.30), indicating fair agreement. Among consultations for which both reviewers initially classified the consultation purpose as diagnostic and both provided an assessment of the study-defined criteria, the agreement for whether consultations met the study-defined criteria was 78.2% (222/284; Cohen’s kappa = 0.50), indicating moderate agreement. Among consultations for which both reviewers judged that the study-defined criteria were not met and both provided a reason classification, the agreement for the factor classification was 50.0% (23/46; Cohen’s kappa = 0.26), indicating fair agreement. The top five specialty departments to which the DGM made diagnostic consultations were the Department of Neurology (12.2%), Rheumatology (11.4%), Gastroenterology (8.5%), Cardiovascular Medicine (8.5%), and Dermatology (7.7%) (Fig. 2). Of the 352 diagnostic consultations, 272 (77.3%) resulted in a correct final diagnosis. For cases resulting in a correct final diagnosis, the median time between the first visit to the DGM and diagnosis was 18.5 days (range, 0-566; IQR 6.5–39). Outpatients had a significantly longer time to diagnosis than did inpatients (mean, 50.9; SD, 91.9 vs. mean, 17.0; SD 15.2, *P* < 0.01).

For the primary outcome, of the 352 cases of diagnostic consultation, 229 cases (65.1%; 95% CI, 59.9–69.9) met the study-defined criteria, and 123 cases (34.9%; 95% CI, 30.1–40.1) did not meet these criteria. There was no statistically significant difference in the proportion of consultations meeting the study-defined criteria between outpatient and inpatient consultations (177/277, 63.9% vs. 52/75, 69.3%; odds ratio [OR] 1.3, 95% CI 0.72–2.3; *P* = 0.38). As shown in Fig. 2, consultations not meeting the study-defined criteria among the top 13 specialty departments with > 10 cases of diagnostic consultations were most noticeable in the Departments of Neurology, Rheumatology, Cardiovascular Medicine, and Urology.

**Fig. 3:**
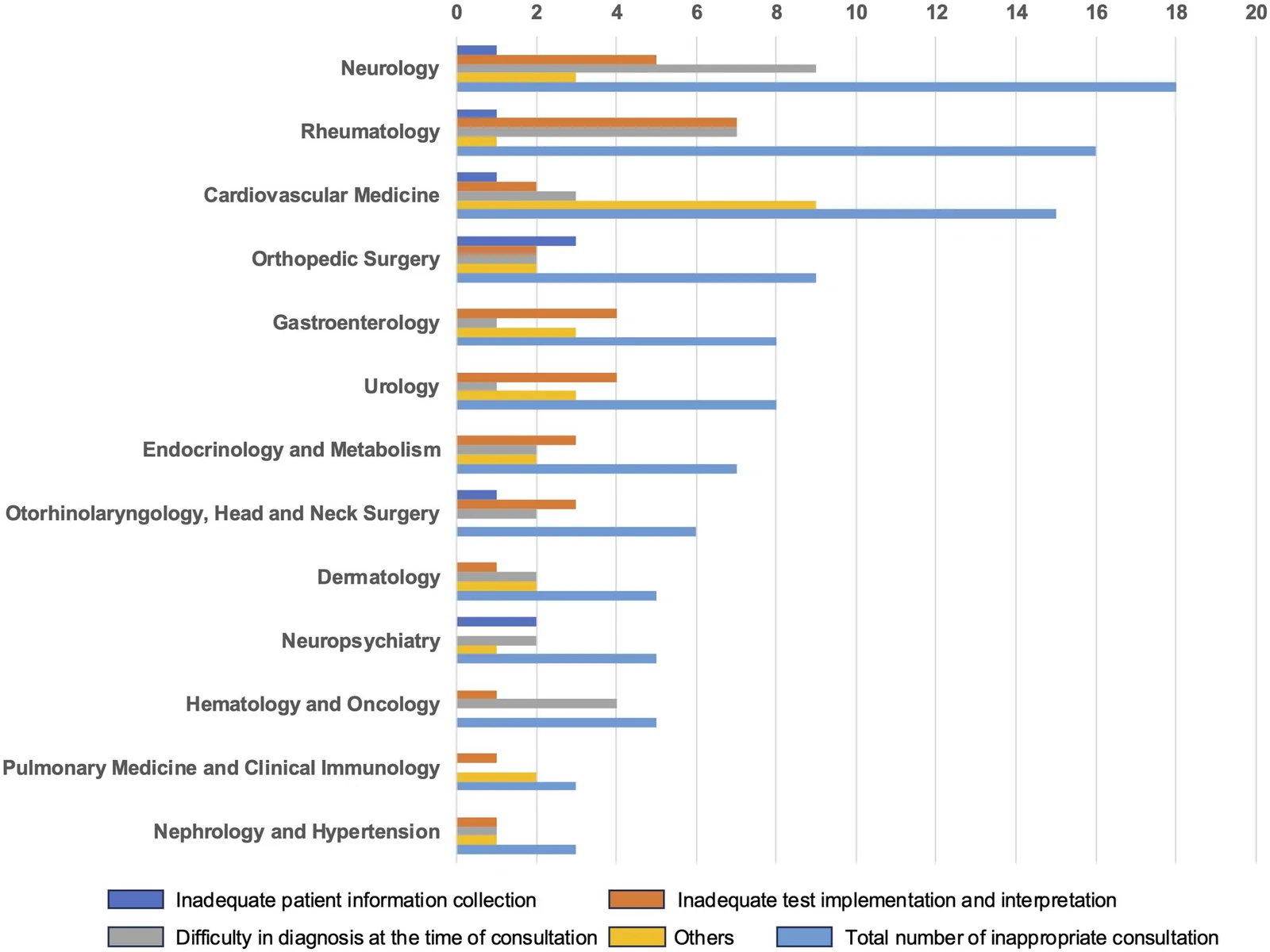
Number of consultations not meeting the study-defined criteria and associated factors among the 13 specialty departments with ≥ 10 diagnostic consultations. The blue scale indicates “inadequate information collection from the patient,” the orange scale indicates “inadequate test implementation and interpretation,” the gray scale indicates “difficult to diagnose at the time of consultation because of undifferentiated symptoms and findings,” and the yellow scale indicates “others”

Factors associated with consultations not meeting the study-defined criteria included inadequate information collection from the patient in 12 cases (9.8%), inadequate test implementation and interpretation in 39 cases (31.7%), difficulty in diagnosis at the time of consultation because of undifferentiated symptoms and findings in 41 cases (33.3%), and others in 31 cases (25.2%) (Table 3). Of the 31 cases classified as others, based on free comments, there were two cases with information integration issues, 13 cases with no comments, and 16 cases that did not meet the study-defined criteria but were considered clinically justified by the reviewers. Figure 3 shows the distribution of the factors associated with consultations not meeting the study-defined criteria from the GIM to the 13 specialty departments with ≥ 10 diagnostic consultations.

**Table 3:** Examples of factors associated with consultations not meeting the study-defined criteria

Factor associated with not meeting the study-defined criteria	Age, Sex	Chief complaint	Specialty department	Purpose	Pre-consultation progress	Post-consultation progress	Explanation for classification as not meeting the study-defined criteria
Inadequate information collection from the patient	30s, F	Chest and back pain	Orthopedic Surgery	Other diagnoses	Chest and back pain lasting approximately 6 months. Cervical spondylosis and thoracic outlet syndrome were considered because the patient had noticed numbness in her left upper limb for 1 month.	Cervical spondylosis and thoracic outlet syndrome were considered negative based on physical findings and radiographic results.	A more detailed physical examination should have been conducted to confirm the absence of neurological abnormalities related to cervical spondylosis and negative findings on provocation tests for thoracic outlet syndrome.
Inadequate information collection from the patient	30s, M	Headache	Neurology	Other diagnoses	No abnormal findings were found on head CT and MRI. Chronic tension headache was suspected based on symptoms.	The diagnostic criteria for primary headaches, including tension headaches, were not met. Psychogenic headache was considered, and medication was started at another hospital.	A more detailed interview revealed that the criteria for chronic tension headaches were unmet, indicating a possibility of psychogenic headaches.
Inadequate implementation and interpretation of the tests	40s, F	Recurrent dizziness	Neurology	Other diagnoses	Head MRI findings indicated neurovascular compression syndrome (NCS) of the facial nerve and cochlear nerve. The possibility that NCS was involved in dizziness was considered.	The involvement of NCS in dizziness was judged to be low. Dizziness was suspected to be auricular vertigo or stress-related.	The consultation was conducted without sufficient consideration of the relationship between the MRI findings of NCS and the dizziness symptoms.
Inadequate implementation and interpretation of the tests	80s, M	Dyspnea	Pulmonary Medicine and Clinical Immunology	Diagnosis with strong suspicion	Chest CT revealed upper lobe emphysema and interstitial pneumonia in the lung bases, with spirometry indicating a reduced FEV1/FVC ratio and diffusion capacity, suggesting combined pulmonary fibrosis and emphysema as a potential cause of dyspnea.	The emphysematous changes were mild, and spirometry results were deemed appropriate for the patient’s age. While chronic interstitial pneumonia was present, its relevance to the symptoms was considered minimal.	The test results had not been interpreted considering the patient’s age.
Difficult to diagnose at the time of consultation	60s, F	Discomfort around both eyes	Neurology	Diagnosis with strong suspicion	No abnormalities in cranial nerve findings or blood tests. Restless face syndrome was suspected.	Considered atypical for restless face syndrome. Poor improvement with pramipexole. Outpatient follow-up with neuropathic pain medications.	Follow-up was conducted in the neurology department; however, a correct final diagnosis was not achieved. As such, based on the study-defined criteria, the consultation was classified as not meeting the criteria.
Difficult to diagnose at the time of consultation	20s, F	Decreased visual acuity	Rheumatology	Other diagnoses	The ophthalmologist diagnosed bilateral branch retinal artery occlusion (BRAO). Painful oral and vulval ulcers were observed, and the possibility of incomplete Behçet disease was considered.	The possibility of incomplete Behçet disease could not be ruled out; however, the possibility was judged to be low as there were no symptoms suggestive of Behçet disease in the past. It was determined that the oral contraceptive was the cause of BRAO.	Specialist follow-up ultimately ruled out the possibility of Behçet’s disease; however, even the specialists could not exclude this possibility at the time of consultation.
Others	70s, F	Chest pain, dyspnea on exertion	Cardiovascular Medicine	Other diagnoses	Chest CT, electrocardiography, and cardiac ultrasonography revealed no abnormalities other than emphysema. Symptoms did not improve with long-acting muscarinic antagonist inhalation. As the patient had cardiovascular risks, consultation for a detailed investigation of coronary artery disease was conducted.	Angina pectoris was considered based on the symptoms, and coronary artery CT and coronary angiography were performed, which showed no abnormal findings. The likelihood of symptoms of cardiac origin was judged to be low.	A correct final diagnosis was not achieved in cardiovascular medicine; therefore, the consultation was classified as not meeting the study-defined criteria. Reviewers’ comment: Given the symptoms and cardiovascular risk, evaluation for coronary artery disease was warranted.
Others	30s, M	Recurrent vomiting	Gastroenterology	Other diagnoses	Recurrent vomiting lasting a year. Blood tests, head CT, contrast-enhanced trunk CT, and upper and lower endoscopy at other hospitals revealed no abnormalities except for intestinal edema at the terminal ileum on CT. It was considered necessary to investigate the small bowel lesion.	No abnormalities were found on small intestine endoscopy. Oral administration of amitriptyline was started for cyclic vomiting syndrome, and the symptoms gradually improved.	A correct final diagnosis was not achieved in gastroenterology; therefore, the consultation was classified as not meeting the study-defined criteria. Reviewers’ comment: None.

Abbreviations: CT, computed tomography; MRI, magnetic resonance imaging; FEV1, forced expiratory volume in 1 second; FVC, forced vital capacity

To assess the robustness of the primary outcome, we performed sensitivity analyses by reclassifying selected subgroups of consultations as meeting the criteria. When the 16 consultations that did not meet the study-defined criteria but were considered clinically justified by the reviewers were reclassified as meeting the criteria, the primary outcome proportion increased from 65.1% (229/352; 95% CI, 59.9–69.9) to 69.6% (245/352; 95% CI, 64.6–74.2). In an exploratory sensitivity analysis reclassifying consultations categorized as difficult to diagnose at the time of consultation because of undifferentiated symptoms and findings as meeting the criteria, the primary outcome proportion increased to 76.7% (270/352; 95% CI, 72.0–80.8). When these diagnostically difficult consultations were excluded from the denominator, the proportion of diagnostic consultations meeting the study-defined criteria was 73.6% (229/311; 95% CI, 68.5–78.2) (Table 4).

**Table 4:** Sensitivity analyses of the proportion of diagnostic consultations meeting the study-defined criteria

Analysis	Consultations meeting criteria, *n*/*N*	Proportion, %	95% CI
Primary analysis	229/352	65.1	59.9–69.9
Reclassifying clinically justified consultations as meeting the criteria	245/352	69.6	64.6–74.2
Reclassifying diagnostically difficult consultations as meeting the criteria	270/352	76.7	72.0–80.8
Excluding diagnostically difficult consultations from the denominator	229/311	73.6	68.5–78.2

There was no statistically significant difference in the proportion of consultations meeting the study-defined criteria between other diagnoses and diagnosis with strong suspicion (146/236, 61.9% vs. 83/116, 71.6%; OR 0.64, 95% CI 0.39–1.1; *P* = 0.07), even after adjusting for age, sex, and location (OR = 0.66, 95% CI 0.40–1.09, *P* = 0.10).

## Discussion

In this study, we found that 57.7% of consultations from the DGM to specialty departments, including outpatients and inpatients, were for diagnostic purposes. Of the diagnostic consultations, 65.1% met the study-defined criteria, indicating that approximately 30% did not meet these outcome-based criteria. These consultations included both potentially modifiable process issues, such as inadequate diagnostic test implementation or interpretation and insufficient patient information, and diagnostically difficult cases with undifferentiated symptoms and findings.

In a previous study in a university hospital’s GIM outpatient department, 36.5% of consultations were considered inappropriate, using an outcome-based definition similar to the study-defined criteria used in the present study [9]. In another study on consultations from the GIM to specialty departments in university hospital outpatient departments, specialists rated 30% of the consultations as inappropriate or potentially inappropriate [10]. Although the definitions and perspectives differed across studies, these findings suggest that a non-negligible proportion of diagnostic consultations may be classified as suboptimal or may not meet predefined criteria, depending on the evaluation perspective.

One-third of diagnostic consultations not meeting the study-defined criteria were identified because of diagnostic difficulties at the time of consultation, which did not lead to new diagnoses during the follow-up period. This was also a major factor in consultations not meeting the study-defined criteria with the Department of Neurology and Rheumatology. Previous studies have indicated that, in half of the cases where consultation with a specialist service was considered but not carried out, no further specialist evaluation or consultation was ultimately required [14, 15]. Furthermore, reports on various symptoms in primary care and fever of unknown origin in hospitals have shown many cases in which symptoms spontaneously improve without a diagnosis [16, 17]. Consultations to specialty departments can be categorized into two types: situations where the need for consultation is clear-cut and situations where there is uncertainty about whether to proceed with consultation [18]. According to previous reports, clear-cut consultation situations are relatively rare in hospital-based GIM departments [18]. In cases of uncertainty regarding whether to consult, strategies such as seeking opinions from colleagues or supervisors and delaying consultation while adopting a watchful-waiting approach to closely monitor the patient, provided there is no urgency, have been proposed [15 ,18]. It has been reported that 15–30% of patients in primary care may present with medically unexplained symptoms, [19] where extensive testing may fail to identify any abnormalities. Additionally, specialty consultations carry the potential risk of overtesting or unnecessary referrals to other specialties due to specialists’ fear of missing conditions within their field of expertise [6]. Given these considerations, to minimize unnecessary consultations, it is crucial to investigate the characteristics of patients who can be safely managed with a watchful-waiting approach. In addition, previous studies on consultations from primary to specialty care have reported that factors that improve the appropriateness of consultations include clear consultation criteria, consultation templates developed with input from the referring primary care physician and specialist, and iterative feedback from the specialist [20–25]. It would be helpful to explore the development of consultation criteria in undifferentiated situations and seek the opinions of specialty departments regarding which cases should be consulted on.

The second most common reason for consultations not meeting the study-defined criteria was inadequate test implementation and interpretation. This finding is consistent with those of previous studies on the causes of diagnostic errors, which reported that problems at the testing phase accounted for the largest number of error factors [26–28]. Based on the results of previous studies, asking colleagues for their opinions before conducting a consultation and regular surveys on consultations not meeting the study-defined criteria may help improve the accuracy of test implementations and interpretations [21, 25].

This study has some limitations. First, the results may not be generalized because this was a single-center, retrospective, observational study conducted in a Japanese university hospital. Although the DGM in this study had roles that are broadly similar to those of many hospital-based GIM departments in Japan, the structure and role of GIM departments vary across institutions and health care systems. Therefore, the findings may not be directly generalizable to settings in which generalists primarily function in community-based primary care or outpatient referral systems rather than as hospital-based generalists. Second, this study retrospectively evaluated diagnostic consultations using operational, outcome-based, study-defined criteria. Therefore, the classification may have been affected by hindsight bias and misclassification [29]. A consultation that did not lead to a correct final diagnosis in the initially consulted specialty department may still have been clinically justified at the time of referral, particularly in cases with undifferentiated, rare, or evolving presentations, or when serious conditions needed to be excluded. Thus, consultations not meeting the study-defined criteria should not be interpreted as clinically inappropriate in all cases. Rather, these consultations should be interpreted as cases that did not meet our outcome-based criteria and may have represented opportunities for improving diagnostic consultation processes. To address this limitation, we performed sensitivity analyses by reclassifying consultations considered clinically justified by the reviewers and by evaluating the impact of consultations categorized as difficult to diagnose at the time of referral. Although the proportion of consultations meeting the study-defined criteria varied across these analyses, the finding that a non-negligible proportion of diagnostic consultations did not meet the outcome-based criteria remained consistent. However, these sensitivity analyses suggest that consultations not meeting the criteria included diagnostically difficult cases that may not necessarily reflect modifiable problems in the diagnostic consultation process. Third, the inter-rater agreement for classifying the purpose of consultations was low, with fair agreement. This may be attributable to the use of six categories for consultation purposes and the difficulty of distinguishing diagnostic consultations from consultations for tests or management in some cases. In addition, agreement for factor classification among consultations not meeting the study-defined criteria was also limited. These findings suggest that the classification of consultation purpose and factors associated with consultations not meeting the study-defined criteria may have limited reproducibility. However, all disagreements were resolved through discussion after re-examining the consultation request, consultation reply, and subsequent clinical course. Future studies should develop more standardized and reproducible criteria for evaluating diagnostic consultations prospectively. Finally, the study-defined criteria differed between “diagnosis with strong suspicion” and “other diagnoses,” because the former required the final diagnosis to be explicitly included in the consultation request. Although this reflected the conceptual difference between a specific suspected diagnosis and a broader diagnostic consultation, it may have affected comparisons between these two diagnostic categories. Therefore, comparisons according to the degree of diagnostic certainty should be interpreted cautiously.

## Conclusion

The present study demonstrated that approximately 30% of diagnostic consultations from GIM to specialty departments did not meet the study-defined criteria. Among these consultations, potentially modifiable factors included inadequate diagnostic test implementation or interpretation and insufficient patient information, whereas approximately one-third involved diagnostically difficult cases with undifferentiated symptoms and findings. Future efforts should focus on improving diagnostic test implementation and interpretation and on identifying cases that can be safely managed by GIM with continued assessment before immediate specialist consultation.

## Data Availability

The datasets used and/or analysed during the current study are available from the corresponding author on reasonable request.

## Abbreviations

GIM: General internal medicine
CIs: Confidence intervals
IQR: Interquartile range
SD: Standard deviation
OR: Odds ratio
